# Salt Cocrystallization—A Method to Improve Solubility and Bioavailability of Dihydromyricetin

**DOI:** 10.3390/pharmaceutics17091209

**Published:** 2025-09-17

**Authors:** Jingping Li, Xinke Chen, Yanan Liu, Caiwu Jiang

**Affiliations:** Institute of traditional Chinese and Zhuang-Yao Ethnic Medicine, Guangxi University of Chinese Medicine, Nanning 530200, China; lijingping2023@stu.gxtcmu.edu.cn (J.L.); chenxinke2023@stu.gxtcmu.edu.cn (X.C.); liuyanan2024@stu.gxtcmu.edu.cn (Y.L.)

**Keywords:** dihydromyricetin, salt cocrystal, solubility, bioavailability

## Abstract

**Objectives**: This study aimed to find salts with similar pharmacological effects designed as cocrystals to improve the aqueous solubility and bioavailability of dihydromyricetin (DMY). **Methods**: A salt-cocrystal solvate (DMY-CIP·C_2_H_6_O) of dihydromyricetin and ciprofloxacin hydrochloride (CIP) was successfully prepared via solvent evaporation method, and further characterized using powder X-ray diffraction, thermal analysis, and infrared spectroscopy. The solubility, stability, bioavailability, and in vitro antimicrobial efficacy of the cocrystal were also studied. **Results**: The cocrystal could increase the solubility of DMY in water and greatly improve the absorption of DMY in vivo (8-fold enhancement in relative bioavailability). In addition, the in vitro antimicrobial efficacy of the cocrystal was comparable to that of CIP, which is a great improvement for DMY. However, due to the formation of cocrystals with salts, the humidity stability of DMY is reduced and it should not be stored in high-humidity environments. **Conclusions**: These findings demonstrate that cocrystallization with water-soluble salts represents an effective strategy for optimizing the pharmaceutical properties of poorly soluble compounds.

## 1. Introduction

The solid-state form of a drug substance fundamentally determines its properties such as dissolution and absorption, thereby significantly influencing therapeutic efficacy. Among various solid forms, crystalline states represent the most prevalent pharmaceutical materials. Conventional crystalline drug forms encompass polymorphs, hydrates, solvates, and salts, and the addition of crystal engineering has led to the concept of pharmaceutical cocrystals [1,2]. Cocrystals are defined as novel crystalline materials formed through non-covalent interactions (e.g., hydrogen bonding, π-π stacking, etc.) between two or more molecular components, exhibiting distinct physicochemical properties [3]. Broadly speaking, cocrystals are classified as multicomponent molecular crystals, including not only anhydrous/non-solvated cocrystals and their polymorphs but also hydrated/solvated cocrystals and their polymorphs, salt cocrystals and their polymorphs, hydrates, and solvates [4,5,6]. These supramolecular assemblies have attracted increasing research attention due to their capacity for molecular-level control over critical drug properties such as stability and solubility [7,8]. Current research on pharmaceutical cocrystals has predominantly focused on the first two categories, whereas salt cocrystals remain relatively underexplored. In this study, a pharmaceutical salt form was selected as the co-crystal former (CCF) with the aim of improving the aqueous solubility and bioavailability of the Active Pharmaceutical Ingredients (API).

**Figure 1:**
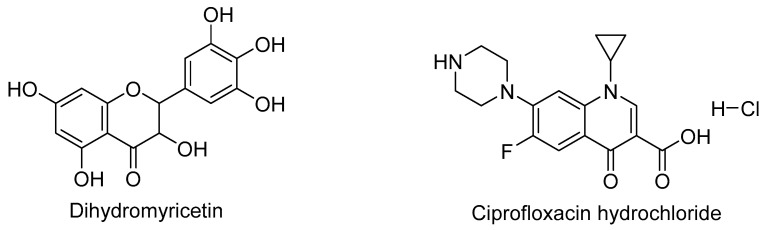
Structural formula of DMY, CIP.

Dihydromyricetin (DMY) is a dihydroflavonol flavonoid compound, whose structure is illustrated in Figure 1. As one of the active constituents of Ampelopsis grossedentata, DMY exhibits a broad spectrum of pharmacological activities, including antioxidant [9], antiviral [10], anti-inflammatory [11], cardiovascular protective [12], antitumor [13], antibacterial [14], alcohol-intoxication alleviating, and hepatoprotective effects [15]. However, its clinical application is limited by low bioavailability. Classified as a Biopharmaceutics Classification System (BCS) class IV drug, DMY possesses both low solubility and low permeability [16]. Therefore, strategies to enhance the druggability of DMY primarily focus on these two aspects. Current research mainly falls into two categories: first, structural modification to improve its lipophilicity and bioactivity [17,18,19]; and second, pharmaceutical formulation technologies such as inclusion complexes, solid dispersions [20], microemulsions [21], micellar systems [22], phospholipid complexes [23], and cocrystals [16,24,25] to enhance its solubility and permeability. Compared to structural modification, which often suffers from low synthetic yield, and solid dispersions, which are associated with stability issues, cocrystal technology offers distinct advantages including good thermodynamic stability, cost-effectiveness, and operational flexibility. Therefore, this study aims to improve the physicochemical properties of DMY through the preparation of pharmaceutical cocrystals.

Conventionally, cocrystals formed with highly soluble coformers tend to exhibit enhanced solubility [26], so most coformers selected for improving cocrystal properties are organic acids, amides, and other substances that have no pharmacological effect and are safe for human bodies. In this study, we deliberately chose pharmacologically active salts as CCF to enhance the aqueous solubility of DMY. Since dihydromyricetin has a slight bacteriostatic effect, it was considered to form a drug–drug cocrystal with ciprofloxacin hydrochloride monohydrate (CIP). CIP represents a first-line fluoroquinolone antibiotic [27] exhibiting broad-spectrum antimicrobial activity, with particular efficacy against Gram-negative bacilli. It is clinically employed for the treatment of urinary tract infections, respiratory infections, and gastrointestinal infections caused by sensitive bacteria. The resulting cocrystal may potentially augment DMY’s antimicrobial efficacy. Its structural formula is shown in Figure 1; the chloride ion in ciprofloxacin hydrochloride serves as an excellent hydrogen bond acceptor, which can easily form intermolecular interactions with the hydroxyl group of DMY. In practical experiments, it was found that DMY and CIP could form cocrystal solvates in various solvents including methanol, ethanol, acetone, and acetonitrile. For safety considerations, only the DMY-CIP ethanol cocrystal solvate was selected for further investigation.

## 2. Materials and Methods

### 2.1. Materials

DMY (purity ≥ 98%) was purchased from Hubei Chenxin Pharmaceutical Co., Ltd. (Wuhan, China), CIP (98% purity) was purchased from Sinopharm Group Chemical Reagents Co., Ltd. (Shanghai, China), methanol (HPLC) was purchased from Thermo Fisher Scientific (China) Co., Ltd. (Shanghai, China), and other reagents were purchased from Sinopharm Group Chemical Reagents Co., Ltd.

### 2.2. Methods

#### 2.2.1. Preparation of DMY-CIP Cocrystal

Equimolar quantities (0.5 mmol each) of DMY and CIP were dissolved in 15 mL of 50% ethanol in a round-bottom flask. The mixture was magnetically stirred at 60 °C for 4 h. A portion of the solution was filtered into a screw-cap bottle, sealed with parafilm containing several pinholes, and allowed to evaporate at room temperature for single crystal growth. The remaining solution was transferred to an open petri dish for bulk cocrystal powder preparation. About one week later, transparent needle-shaped crystals were obtained.

#### 2.2.2. Powder X-Ray Diffraction (PXRD)

Powder X-ray diffraction analysis was performed using a Rigaku MiniFlex 600 diffractometer (Rigaku Corporation, Tokyo, Japan) with Cu Kα radiation (*λ* = 1.54059 Å) at 40 kV and 150 mA. The samples were scanned from 3° to 50° (2θ) with a step size of 0.01° and a scanning rate of 10°/min. An appropriate amount of powder sample was ground and placed on a monocrystalline silicon sample stage, and the sample was flattened using a medicine spoon and weighing paper for testing. Data analysis was conducted using Origin 2021 software, and single-crystal powder diffraction patterns were simulated with Mercury 2021.3.0 software.

#### 2.2.3. Thermogravimetric (TG) Analysis and Differential Scanning Calorimetry (DSC)

Thermal analysis was performed using a Netzsch STA449 F5 instrument (Netzsch-Gerätebau GmbH, Selb, Germany). Samples (2–5 mg) were weighed into alumina crucibles and heated from 30 °C to 800 °C at a constant heating rate of 10 °C/min under a dynamic nitrogen atmosphere. The protective gas (N1) and purge gas (N2), both high-purity nitrogen, were maintained at flow rates of 5 mL/min and 20 mL/min, respectively.

#### 2.2.4. Fourier Transform Infrared Spectroscopy (FTIR)

Infrared spectra were acquired using a PerkinElmer Spectrum Two spectrometer (PerkinElmer Inc., Springfield, IL, USA) in the range of 4000–500 cm^−1^ with a resolution of 4 cm^−1^. Samples were prepared by thoroughly grinding with dried potassium bromide and pressing into sheets. The final spectra represent averages of 32 consecutive scans.

#### 2.2.5. Single-Crystal X-Ray Diffraction (SCXRD)

Single-crystal X-ray diffraction data were collected at 293 K using a Bruker Apex Duo diffractometer (Bruker Corporation, Bremen, Germany) with Cu Kα radiation (*λ* = 1.54178 Å). The structure was solved with the Olex2-1.5 solve structure solution program using Charge Flipping and refined with the SHELXL [28] refinement package using Least Squares minimization.

#### 2.2.6. High-Performance Liquid Chromatography (HPLC)

Chromatographic analysis was performed using an Agilent 1260 HPLC (Agilent Technologies Inc., Santa Clara, CA, USA) The chromatographic separation was performed on an Acclaim C18 reversed-phase column (5 μm, 120 Å, 4.6 × 250 mm). The mobile phase consisted of methanol–0.1% formic acid aqueous solution (40:60, *v*/*v*) at a flow rate of 1.0 mL/min. The column temperature was maintained at 30 °C, with detection wavelength set at 291 nm and injection volume of 10 μL. Samples were dissolved in 50% methanol prior to analysis. Under these conditions, complete resolution between DMY and CIP was achieved.

#### 2.2.7. Solubility and Dissolution Experiments

Solubility experiments: Excess amounts of DMY and DMY-CIP powder were placed separately in screw-cap bottles containing 5 mL deionized water. The sealed bottles were equilibrated in a temperature-controlled magnetic stirrer at 20 °C, 25 °C, 30 °C, 35 °C, and 40 °C with continuous stirring at 200 rpm for 24 h. After equilibration, aliquots were filtered and appropriately diluted with 50% methanol for HPLC analysis of DMY content. All experiments were performed in triplicate. The remaining powder was filtered and dried at the end of the experiment and tested for PXRD.

Dissolution Study: Owing to the excessively rapid dissolution of DMY and its cocrystal under sink conditions, which precludes a meaningful comparison of their dissolution behaviors, this study employed non-sink conditions to investigate the dissolution characteristics of different solid forms. The detailed experimental procedure was as follows: Two 20 mL aliquots each of hydrochloric acid buffer (pH 1.2) and phosphate buffer (pH 6.8) were prepared. To one aliquot of each buffer, a 0.5% CMC-Na solution was added to achieve a final concentration of 0.1% (*w*/*v*) CMC-Na. These prepared media were placed in round-bottom flasks, which were then equilibrated at 37 °C for 30 min in a thermostatic magnetic stirrer. An excess amount of DMY or DMY-CIP powder was subsequently introduced into each flask, and the dissolution test was initiated under constant stirring at 240 rpm and 37 °C. Samples were withdrawn at predetermined time intervals (5, 10, 20, 30, 45, 60 min, 2, and 4 h), immediately filtered through a 0.45 μm membrane filter, and the withdrawn volume was replaced with an equal volume of fresh pre-warmed medium. The filtered samples were appropriately diluted, and a 10 μL aliquot was injected for High-Performance Liquid Chromatography (HPLC) analysis. All experiments were performed in triplicate. After continuous stirring for 24 h, the residual solids were collected and subjected to crystal form identification by X-ray Powder Diffraction (XRPD).

#### 2.2.8. Stability Test

Stability evaluations were conducted by subjecting appropriate quantities of the raw material and cocrystal powder to various stress conditions: thermal stability was assessed by storage at 40 °C for 3 months and 60 °C for 10 days, while hygroscopic stability was examined under 75% RH for 1 month and 92.5% RH for 7 days. The hygroscopicity was quantitatively determined gravimetrically, with the percentage weight increase calculated as [(Wt − W0)/W0] × 100%, where W0 and Wt represent the initial and final weights, respectively. Following stability testing, samples were visually inspected for physical changes, analyzed by HPLC for content variation, and characterized by PXRD to evaluate crystalline form stability, thereby assessing both physical and chemical stability.

#### 2.2.9. Bioavailability

The physical mixture and cocrystal samples were suspended in 0.5% CMC-Na solution to prepare 10 mg/mL gavage formulations as a gavage-administered preparation. Twelve male SD rats were randomly divided into two groups (*n* = 6) after 5-day acclimatization feeding. The rats were fasted for 12 h before the experiment (free access to water) and were administered by gavage at a dose of 150 mg/kg within the safe [29] and effective dose range. Blood samples (0.5 mL) were collected from the eye sockets at 0.083 h, 0.25 h, 0.5 h, 0.75 h, 1 h, 1.5 h, 2 h, and 3 h, respectively, in heparinized centrifuge tubes. The plasma was separated by centrifugation (10,000 rpm, 10 min) and saved at −4 °C until analysis.

The plasma was treated by liquid–liquid extraction: 200 μL of drug-containing plasma was accurately measured and placed in a 10 mL plugged centrifuge tube, followed by adding 30 μL of methanol, 10 μL of internal standard working solution (luteolin, 5 μg/mL), and 400 μL of 0.1% acetic acid solution, vortex mixing for 1 min. Four mL ethyl acetate was added as the extraction solvent, vortex extracted for 2 min, and centrifuged at 4200 rpm for 10 min. The supernatant was transferred and dried by nitrogen at 40 °C in a water bath. The residue was redissolved with a 150 μL mobile phase. After centrifugation, 20 μL supernatant was taken for HPLC analysis. The pharmacokinetic parameters were calculated by DAS 2.0 software based on a non-compartmental model.

#### 2.2.10. Antibacterial Activity Assay

In this study, the in vitro antibacterial activity of DMY, CIP, physical mixtures, and cocrystals against Escherichia coli and Staphylococcus aureus was evaluated by the filter paper diffusion method. In this experiment, 6 mm-diameter sterile filter papers were used to load 10 μL 10% ethanol, dihydromyricetin solution (0.1 mg/mL), ciprofloxacin hydrochloride solution (0.1 mg/mL), mixed solution, and cocrystal solution (equivalent to DMY 0.1 mg/mL), respectively. The filter papers were pasted on an agar plate uniformly coated with 100 μL bacterial suspension (108 CFU/mL). After 18–24 h of culture at 37 °C, the diameter of the inhibition zone was accurately measured.

The MIC values were determined using the standard broth microdilution method in 96-well plates according to CLSI guidelines. All procedures, including preparation of drug solutions, bacterial suspensions, serial two-fold dilutions, and inoculation, were performed under aseptic conditions. Bacterial suspensions (approximately 10^6^ CFU/mL) were added to wells containing serially diluted drug solutions, followed by incubation at 37 °C for 18–24 h. The MIC was initially assessed visually as the lowest drug concentration showing complete inhibition of visible growth (clear wells). For quantitative confirmation, the optical density at 600 nm (OD_600_) was measured using a microplate reader, with an OD variation threshold of <0.05 defining the MIC. All experiments were performed in triplicate, and the results were expressed as mean values.

## 3. Results

### 3.1. Powder X-Ray Diffraction

**Figure 2:**
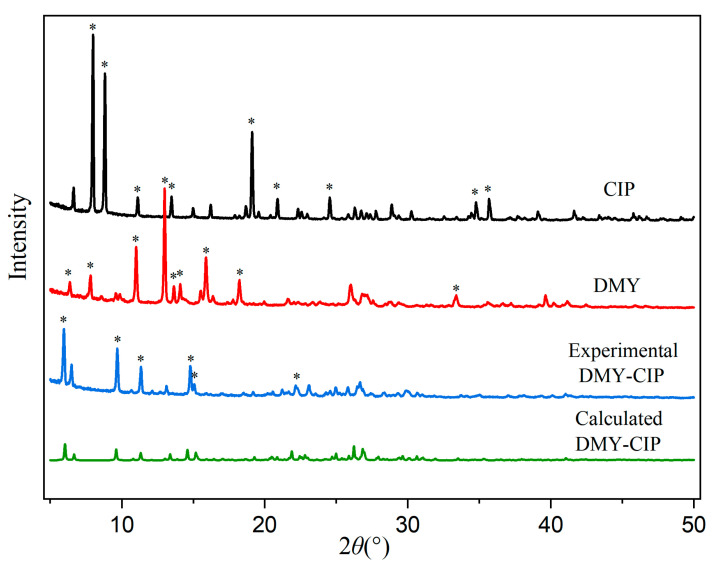
PXRD patterns of DMY, CIP, and DMY-CIP cocrystal. Hexagram symbol indicates diffraction peaks.

The powder X-ray diffraction patterns of the starting materials and cocrystal are presented in Figure 2. Characteristic diffraction peaks corresponding to dihydromyricetin at 6.37°, 7.82°, 11.01°, 13.00°, 13.64°, 14.09°, 15.97°, 18.23°, and 33.39°, as well as those of ciprofloxacin hydrochloride at 7.99°, 8.82°, 11.11°, 13.48°, 19.11°, 20.89°, 24.54°, 34.76°, and 35.68° were absent in the cocrystal pattern. Instead, the cocrystal exhibited distinct new diffraction peaks at 5.94°, 9.69°, 11.34°, 14.79°, 15.08°, and 22.16°, which differed from those of each API, confirming successful cocrystal formation. The experimental PXRD pattern showed agreement with the simulated pattern derived from single-crystal data, indicating high crystalline purity of the obtained cocrystal.

### 3.2. Thermal Analysis

**Figure 3:**
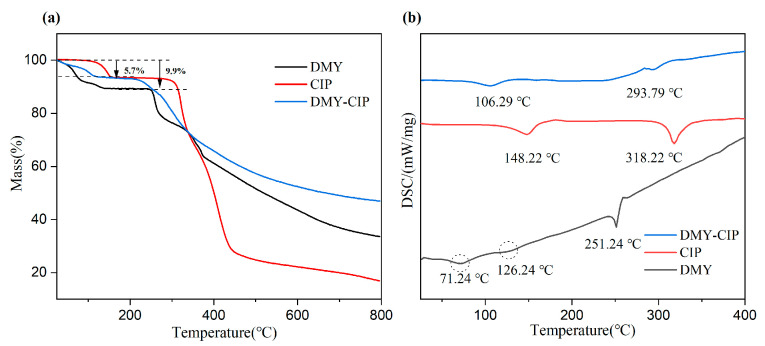
TG (**a**) and DSC (**b**) of DMY, CIP, and DMY-CIP cocrystal.

The DSC and TG diagrams of APIs and cocrystals are shown in Figure 3. Dihydromyricetin exhibited three endothermic peaks in its thermal analysis profile. The first two endothermic events observed at 71.24 °C and 126.24 °C corresponded to weight losses of 5.7% and 9.9% in the thermogravimetric analysis, respectively, indicating the sequential loss of two water molecules and confirming its dihydrate nature. The third endothermic peak at 251.24 °C represented the melting point of DMY, with concomitant thermogravimetric analysis demonstrating immediate decomposition following melting. CIP contains a crystal water. The endothermic peak at 148.22 °C in the DSC diagram and the first weight loss platform in the TG diagram prove that the crystal water is lost, and 318.22 °C is the melting point of CIP, which is consistent with the literature report [30]. The endothermic peak of the cocrystal at 106.29 °C corresponds to the weight loss platform in the TG curve, which confirms the removal of ethanol molecules in the cocrystal. The melting point of the cocrystal is 293.79 °C, which is between the two raw materials. This characteristic transition temperature confirms the formation of the new crystal phase.

### 3.3. Fourier Transform Infrared Spectroscopy Analysis

**Figure 4:**
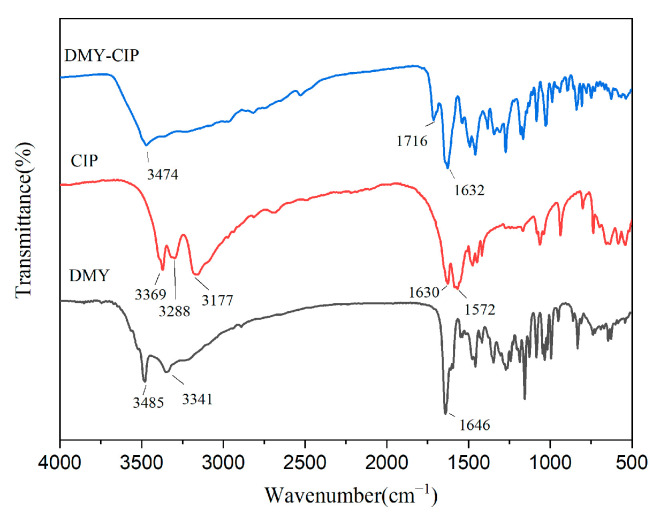
Infrared spectra of DMY, CIP, and DMY-CIP cocrystals.

The infrared spectra of raw materials and cocrystals are shown in Figure 4. The characteristic peaks of DMY at 3485 cm^−1^ and 3341 cm^−1^ are O-H stretching vibration. The characteristic peak at 1646 cm^−1^ corresponds to the stretching vibration of C=O. The characteristic peaks of CIP appeared at 1630 cm^−1^ and 3177 cm^−1^, which represented the characteristic absorption bands of O-H and C=O in the carboxyl group, respectively. The characteristic peak at 3288 cm^−1^ represents the stretching vibration of N-H. After cocrystal formation, the C=O stretching vibration peak of CIP was shifted to 1716 cm^−1^, and the O-H stretching vibration peak of dihydromyricetin was shifted to 3474 cm^−1^. These spectral peak shifts reflect that the hydrogen bond mode of the raw material changes with the formation of the cocrystal.

### 3.4. Crystal Structure Analysis

**Figure 5:**
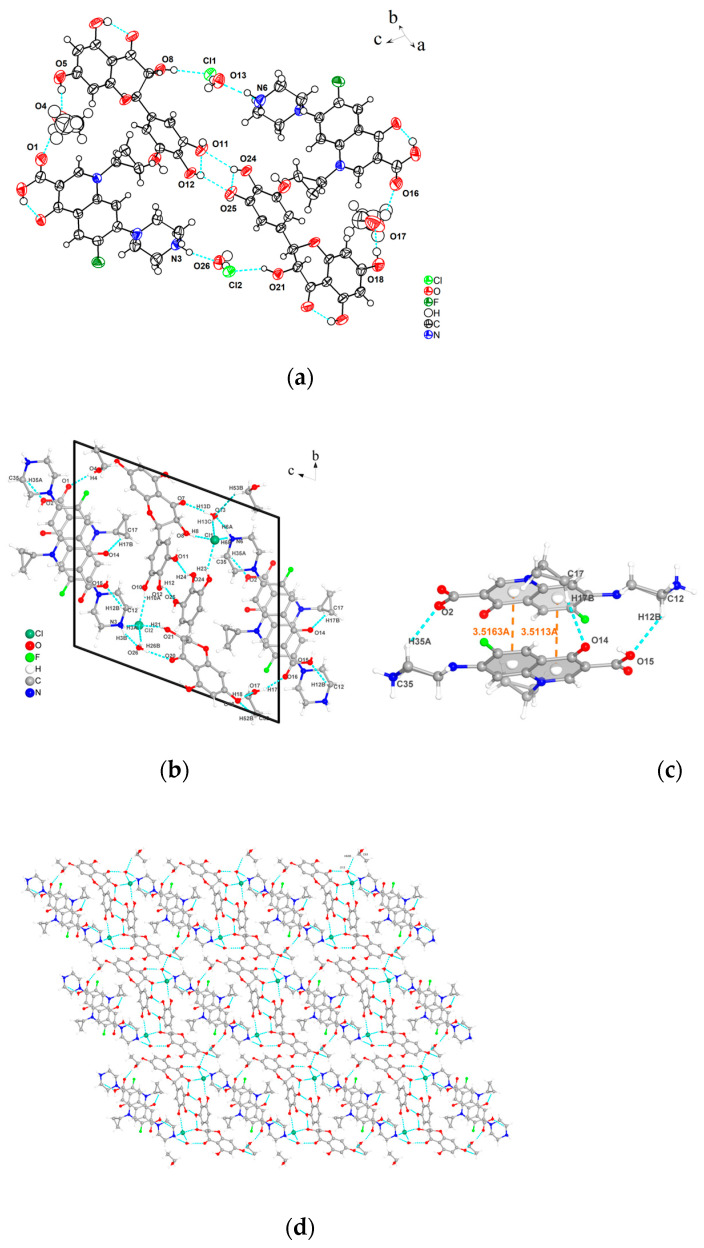
(**a**) Asymmetric unit, (**b**) individual cell diagram, (**c**) π-π stacking and hydrogen bonds of CIP, (**d**) 3D stacking diagram of the a-axis.

Before single crystal diffraction, the crystal was determined to be a cocrystal with a stoichiometric ratio of 1:1 by HPLC. The obtained crystal structure is shown in Figure 5, The 3D stacking diagrams of the b-axis and c-axis are shown in Figure S1 of the supplementary materials. and the crystallographic data are presented in Table 1. DMY-CIP·C_2_H_6_O belongs to the P1 space group in the triclinic system, and the asymmetric unit includes two DMY molecules, two CIP molecules, and two ethanol molecules. CIP exists in the form of monohydrate hydrochloride (proton exchange between Cl^−^ and N^+^ ions), and water molecules are connected to ciprofloxacin by hydrogen bonds O13-H13C···Cl1, N6-H6A···O13. DMY molecules are connected to CIP molecules by hydrogen bonds through chloride ions and ethanol molecules to form a tetramer, while the two DMY molecules are connected by hydrogen bonds O24-H24···O11, O12-H12···O25 to form a dimer. The DMY-CIP cell diagram is shown in Figure 5b; the CIP molecules are connected by π-π stacking and hydrogen bonds (C35-H35A···O2, C17-H17B···O14, C12-H12B···O15). The connected CIP molecules are also linked to the corresponding DMY molecules to form octamers, and together these molecules form a crystal cell. In addition, the water molecules are hydrogen-bonded to the ethanol molecules via C53-H53B···O13 and stacked toward the b-axis, forming a three-dimensional stacking structure.

In crystal engineering, a fundamental design principle involves the hypothesis that hydrogen bond interactions adhere to the “best donor-best acceptor” rule [31]. According to Table 2, the O-H···O motif represents the strongest hydrogen-bond interaction (with shorter bond lengths), followed by O-H···Cl. In the DMY-CIP cocrystal, oxygen atoms and chloride ions serving as the primary hydrogen-bond acceptors consistently interact with the strongest H-bond donors, namely the O-H groups. These strong O-H donors originate from both DMY and solvent molecules. Specifically, the ethanol molecule serves as a “bridge” between the API and the coformer, while also occupying the voids created by the geometric mismatch between the API and coformer, thereby improving spatial packing and enhancing cocrystal stability. Consequently, the incorporation of DMY and the solvent modifies the original coordination environment of CIP [32], leading to the formation of stronger hydrogen-bonding patterns. This observation is further supported by the cocrystallization process, where modifications in experimental conditions (including temperature, agitation rate, reaction duration, and solvent ratios) consistently yield the cocrystal phase, demonstrating to some extent the robustness of this solid form. Naturally, these structural modifications lead to altered physicochemical properties of CIP, such as reduced solubility, which will be experimentally verified in subsequent sections.

### 3.5. Hirshfeld Surface Analysis

**Figure 6:**
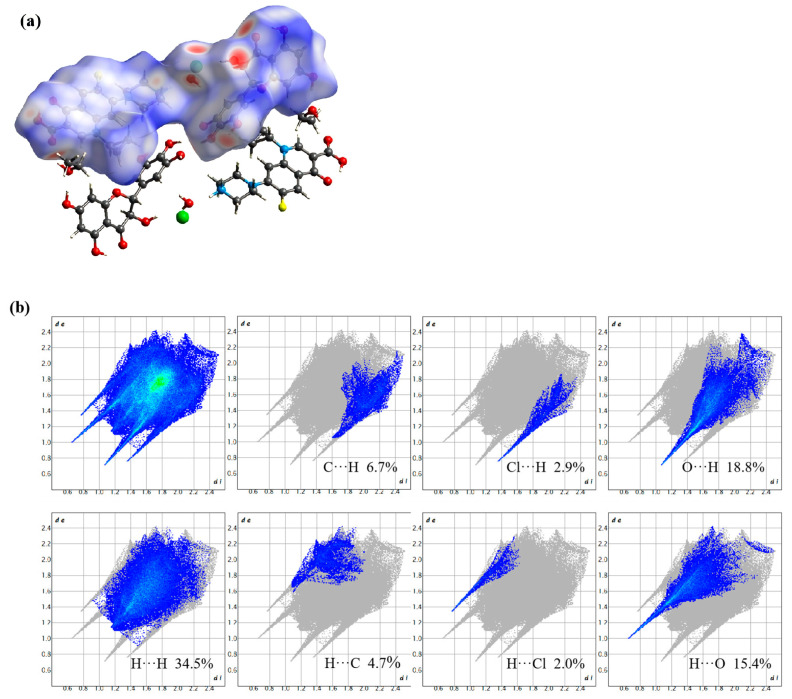
DMY-CIP mapping of Hirshfeld surfaces on d_norm_ surfaces (**a**) and 2D fingerprint maps (**b**).

Hirshfeld surface analysis serves to visualize and quantify intermolecular interactions within molecular crystal structures [33]. Specifically, red regions on the mapped Hirshfeld surfaces typically indicate strong attractive interactions, such as hydrogen or strong halogen bonds, while blue areas correspond to van der Waals contacts or C-H···π interactions. The associated two-dimensional fingerprint plots further delineate the nature and extent of these interactions [34,35]. The lower left region of the plot corresponds to strong interactions, the upper right to weak contacts, and hydrogen bonds typically appear as a pair of sharp spikes pointing toward the bottom left. Additionally, fingerprint plots allow quantitative comparison of the relative contributions of various interaction types. As shown in Figure 6, the primary hydrogen bond acceptors in the cocrystal are oxygen atoms and chloride ions. The O···H, H···O, Cl···H, and H···Cl interactions appear in the corner regions of the fingerprint plot, with contribution rates of 18.8%, 15.4%, 2.9%, and 2.0%, respectively. The O···H and H···O interactions exhibit numerous sharp spikes closer to the origin (de, di ≈ 0), indicating that these are the dominant conformational forces stabilizing the DMY-CIP cocrystal structure. In contrast, H···H, C···H, H···C, and C···C interactions collectively contribute more than 50% (34.5%, 6.7%, 4.7%, and 6.4%, respectively), yet are located near the center of the fingerprint plot, suggesting they serve mainly as structural packing forces without directing specific conformational preferences.

### 3.6. Solubility and Dissolution Analysis

**Figure 7:**
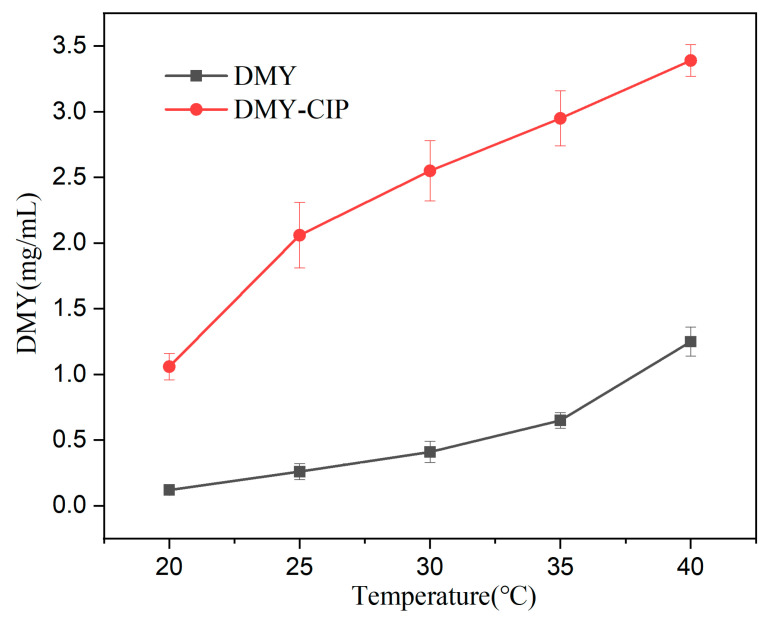
Water solubility of DMY and DMY-CIP at different temperature.

The water solubility curves of DMY and DMY-CIP with temperature changes are shown in Figure 7. The solubility of DMY in cocrystals is much higher than that of raw material in the tested temperature band, ranging from 2–8 times higher. Since the solubility of DMY all increases with temperature, the solubility advantage of cocrystals is more obvious in the low temperature region. Correspondingly, cocrystallization markedly reduced the water solubility of CIP from the original 38.4 mg/mL [36] to about 3.2 mg/mL (T = 303.15 K), which can be attributed to the strengthened hydrogen bonding interactions between ionic groups in the cocrystal (including the formation of an extensive 3D hydrogen bonding network), as mentioned above.

**Figure 8:**
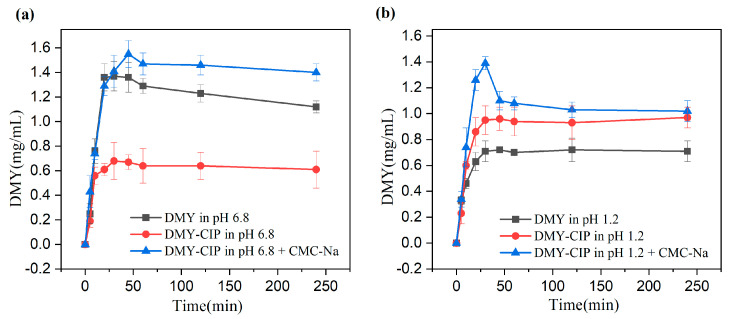
(**a**) Dissolution of DMY and DMY-CIP in pH 6.8 buffer solution, (**b**) dissolution of DMY and DMY-CIP in pH 1.2 buffer solution.

The dissolution curves at different pHs are shown in Figure 8. The dissolution trends of cocrystals and DMY are similar, and the two systems tend to balance after reaching the peak quickly. In pH 1.2 buffer solution, the cocrystal showed a better dissolution degree and speed, reaching an equilibrium solubility of about 1 mg/mL compared with 0.7 mg/mL of DMY. The addition of CMC-Na further enhanced the dissolution advantage of the cocrystal, and the solubility of DMY in the cocrystal system was increased to 1.4 mg/mL. However, the dissolution advantage of the eutectic at pH 6.8 disappeared. A possible reason is that CIP mainly exists in the form of zwitterions at pH 6.8 (pKa1 ≈ 6.1, pKa2 ≈ 8.7). The decrease in electrostatic repulsion is beneficial to aggregation. The insoluble CIP ± impairs the dissolution of DMY through the limitation of the solubility product, resulting in lower apparent solubility of DMY within the cocrystal than its standalone form. However, the addition of CMC-Na reversed this phenomenon. The possible mechanism is that CMC-Na increases the viscosity of the solution, hinders the free movement and collision of drug molecules, and inhibits crystal nucleation. Secondly, the long-chain macromolecules of CMC-Na can be adsorbed on the surface of the newly formed crystal, and the crystal growth is inhibited by the steric hindrance effect. Thus, the supersaturation state is stabilized, showing a higher apparent solubility. After the solubility and dissolution experiments, the crystal phase of the remaining solid was tested. The results showed that the crystal phase of the cocrystal did not change in water and pH 1.2 buffer, indicating that the hydrogen bond was relatively stable. The change of crystal form of cocrystal in pH6.8 solution may be caused by the instability of DMY in pH 6.8 solution. The PXRD pattern is shown in Figure S2 of the supplementary material.

### 3.7. Stability Analysis

**Figure 9:**
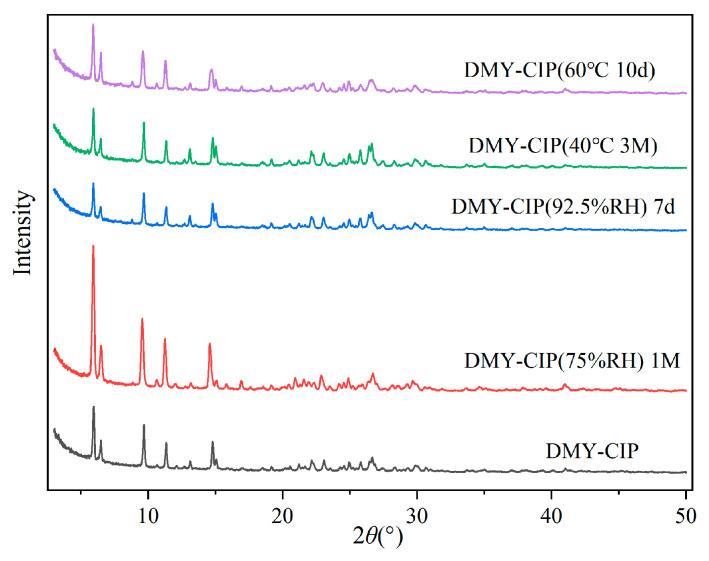
Crystallographic changes of eutectics under high-temperature and high-humidity conditions.

The crystal form changes of the DMY-CIP are shown in Figure 9, the content changes are presented in Table 3, and the hygroscopicity is shown in Table 4. The cocrystal powder maintained good stability under high-temperature conditions, with no observable changes in physical appearance, crystalline form, or drug content compared to the initial sample. The cocrystal maintains physical crystalline stability under high-humidity conditions, but exhibits reduced chemical stability. Due to the inherent hygroscopic properties of hydrochloride, the hygroscopicity of DMY-CIP was significantly higher than that of DMY raw material in a 92.5% RH environment, but the difference between the two was not obvious in 75% RH conditions. As for CIP, cocrystallization greatly reduces its hygroscopicity in high-humidity environments, which is attributed to the formation of hydrogen bonding between chloride ions and DMY, thus reducing the chance of forming hydrogen bonding with water molecules. In addition, DMY is easily oxidized in high-humidity environments, which is aggravated by cocrystals. After a month of storage at 75% RH, the content of DMY in cocrystal decreases by about 4%, which is not as stable as that of raw materials.

### 3.8. Bioavailability Analysis

**Figure 10:**
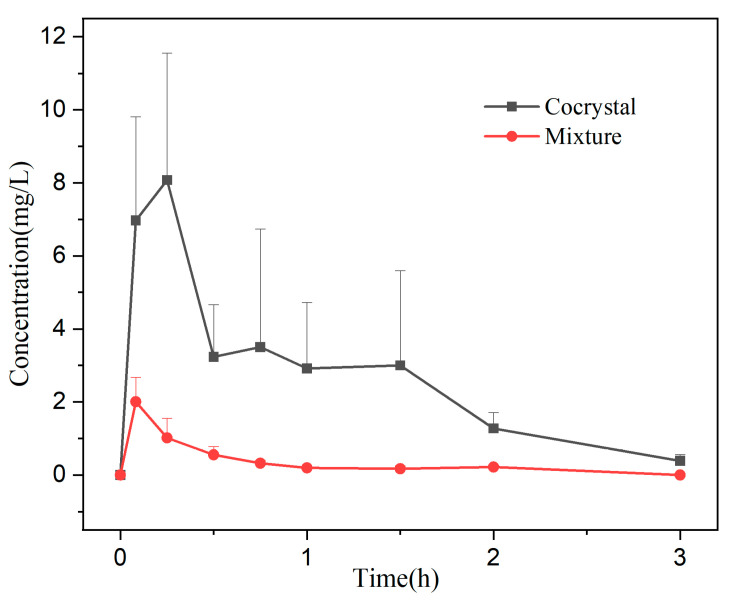
Pharmacokinetic curves of the mixture and the cocrystal.

The plasma concentration–time curve of the mixture and cocrystal are shown in Figure 10. The trend of DMY blood concentration in the mixture and cocrystal groups was to reach C_max_ in a short period of time, and then it was eliminated rapidly in the body, and DMY was essentially undetectable in the blood after 3 h. Pharmacokinetic parameters are summarized in Table 5. As shown, the cocrystal group demonstrated significantly higher plasma concentrations at all time points compared to the mixture group, with an enhancement ranging from approximately 2–10-fold. Specifically, the AUC_(0-t)_ of the cocrystal was about nine times greater, and its C_max_ was approximately 4.8 times higher than that of the mixture, indicating a great improvement in DMY absorption. The half-life of the cocrystal increased slightly, but the difference was not statistically significant (*p* > 0.05). Combined with the in vitro dissolution results, the cocrystal exhibited superior absorption characteristics in vivo, which may be attributed to the addition of CMC-Na. The homogeneous suspension of the DMY-CIP cocrystal in the CMC-Na solution prevented powder aggregation and ensured favorable dispersion in vivo. Moreover, the inclusion of CMC-Na likely contributed to the inhibition of crystal growth and helped maintain a supersaturated state of the solution [37,38], collectively enhancing the bioavailability of the cocrystal. In contrast, the effect of CMC-Na on DMY is much smaller, limited by inherent solubility and stability [39]; DMY reaches peak blood concentration at 5 min, after which the rate of elimination is much greater than the rate of absorption, and it is rarely present in the bloodstream.

### 3.9. In Vitro Antimicrobial Activity

The diagram of the antibacterial zone is shown in Figures S3 and S4 of the supplementary materials. From Table 6, Table 7, it can be seen that DMY has a higher growth inhibition threshold against *S. aureus* and *E. coli* and needs higher concentration to produce a zone of inhibition. The CIP, physical mixture, and cocrystal showed significant inhibition against *E. coli* and *S. aureus*, and the inhibition effect against *E. coli* was better. The difference in the antibacterial effects of the three drugs was not significant (*p* > 0.05), indicating that the addition with dihydromyricetin did not affect the antimicrobial capacity of CIP, nor did it increase its effect. However, for DMY, the cocrystal as a whole improved the antibacterial ability.

## 4. Discussion

This work enhanced the solubility and bioavailability of dihydromyricetin by forming salt eutectics. There are still two points worthy of attention. One is that the cocrystal reduces the humidity stability of DMY, and the other is that it reduces the solubility of CIP. Regarding the first point, subsequent formulation studies will consider incorporating low-hygroscopicity diluents such as anhydrous lactose or hydrophobic polymers like polyethylene glycol to mitigate this issue, along with storage under dry conditions to minimize moisture exposure. Concerning the second point, although reduced solubility of CIP may lead to decreased bioavailability, this is not entirely disadvantageous. Lower solubility can facilitate sustained release, suggesting potential for the development of extended-release formulations. Moreover, the moderate reduction in solubility supports more uniform drug delivery and may enhance medication safety. Additionally, beyond its hepatoprotective effects, DMY exhibits beneficial health properties; some studies indicate its ability to modulate the composition and abundance of gut microbiota [40,41], which may help alleviate certain side effects associated with CIP.

Overall, this study achieved its objectives, but there remains room for improvement. Previous studies on cocrystal solubility advantages indicate that such enhancements primarily result from the generation of a supersaturated state. Since cocrystals eventually dissociate into their individual components in solution, relying solely on cocrystallization to improve drug absorption may be insufficient. Thus, implementing formulation and processing strategies to maintain supersaturation is of critical importance. Dissolution and bioavailability tests confirmed that CMC-Na helps sustain the supersaturated state of DMY, but other polymers such as PVP and HPC were not evaluated. Furthermore, the current study focused solely on solubility; another key factor influencing bioavailability—permeability—was not investigated. Both the cocrystal itself and potential excipients may influence the permeability of DMY. Therefore, future studies should focus on screening auxiliary agents and systematically evaluating their effects on solubility and permeability. Lastly, the low bioavailability of DMY may also be attributed to its inherent instability. Follow-up development of solid dosage forms may consider employing microencapsulation or inclusion complexes to enhance stability, which could simultaneously address the reduced humidity stability observed in the cocrystal.

## 5. Conclusions

In summary, a salt cocrystal (DMY-CIP) containing one ethanol molecule was successfully prepared via te solution evaporation method, with a 1:1 stoichiometric ratio between DMY and CIP. Comprehensive characterization was performed using X-ray diffraction, thermal analysis, and Fourier transform infrared spectroscopy. The two drug molecules in the cocrystal are interconnected via chloride ions and ethanol molecules. Stability, solubility, and bioavailability evaluations demonstrated that DMY-CIP formation reduced the hygroscopicity of CIP while improving the solubility and bioavailability of DMY. Moreover, the cocrystal exhibited comparable in vitro antibacterial efficacy to CIP. These findings suggest that poorly soluble compounds can enhance their solubility and oral absorption through salt cocrystallization.

## Figures and Tables

**Table 1:** Crystallographic data of the pharmaceutical cocrystal.

Crystal Parameter	DMY-CIP
Empirical formula	C34H39ClFN3O13
Formula weight	752.13
Temperature/K	293 (2)
Crystal system	triclinic
Space group	P1
a/Å	6.89700 (10)
b/Å	15.8699 (3)
c/Å	16.6818 (4)
α/°	69.526 (2)
β/°	88.418 (2)
γ/°	80.1800 (10)
Volume/Å3	1684.48 (6)
Z	2
ρcalcg/cm^3^	1.483
μ/mm^−1^	1.697
F(000)	788.0
Crystal size/mm^3^	0.21 × 0.16 × 0.13
Radiation	CuKα (*λ* = 1.54178)
2θ range for data collection/°	9.616 to 140.138
Index ranges	−6 ≤ h ≤ 8, −19 ≤ k ≤ 19, −19 ≤ l ≤ 20
Reflections collected	20,356
Independent reflections	8702 [Rint = 0.0388, Rsigma = 0.0412]
Data/restraints/parameters	8702/27/968
Goodness-of-fit on F2	1.077
Final R indexes [I >= 2*σ* (I)]	R1 = 0.0425, wR2 = 0.1210
Final R indexes [all data]	R1 = 0.0472, wR2 = 0.1255
Largest diff. peak/hole/e Å-3	0.49/−0.48
Flack parameter	−0.001 (17)

**Table 2:** Hydrogen bond parameters of the pharmaceutical cocrystal.

D	H	A	d (D–H)/Å	d (H–A)/Å	d (D–A)/Å	D–H–A/°
O12	H12	O25	0.82	2.05	2.713 (4)	137.2
O8	H8	Cl1	0.82	2.27	3.078 (3)	167.0
O24	H24	O11	0.82	2.24	2.966 (4)	147.2
O10	H10A	Cl2	0.82	2.40	3.127 (3)	148.5
O13	H13C	Cl1	0.85	2.28	3.105 (4)	162.5
O13	H13D	O7	0.85	1.97	2.820 (5)	176.1
O23	H23	Cl1	0.82	2.46	3.207 (3)	151.0
O18	H18	O17	0.82	1.79	2.611 (6)	174.0
O5	H5	O4	0.82	1.83	2.643 (7)	171.9
N6	H6A	O13	0.89	1.83	2.703 (5)	168.4
N6	H6B	Cl1	0.89	2.24	3.123 (4)	169.5
N3	H3A	Cl2	0.89	2.23	3.114 (4)	169.2
N3	H3B	O26	0.89	1.85	2.722 (5)	165.2
C17	H17B	O14	0.97	2.57	3.516 (6)	165.4
C12	H12B	O15	0.97	2.50	3.304 (6)	139.7
O4	H4	O1	0.82	1.89	2.694 (7)	166.0
O17	H17	O16	0.82	1.95	2.685 (7)	148.3
C35	H35A	O28	0.97	2.51	3.309 (6)	139.3
C52	H52B	O18	0.96	2.56	3.409 (15)	147.2
C53	H53B	O13	0.97	2.58	3.359 (12)	137.3
O21	H21	Cl2	0.95 (5)	2.16 (5)	3.043 (3)	154 (4)
O26	H26A	Cl2	0.90 (7)	2.23 (7)	3.085 (4)	157 (6)
O26	H26B	O20	0.76 (11)	2.10 (11)	2.855 (5)	174 (12)

**Table 3:** Content of DMY and cocrystal under different conditions.

Sample	The Content of DMY (%)				
	Normal	40 °C	60 °C	75% RH	92.5% RH
DMY	97.63 ± 0.07	97.51 ± 0.03	97.35 ± 0.1	95.41 ± 0.06	97.33 ± 0.08
DMY-CIP	97.54 ± 0.12	97.50 ± 0.05	97.59 ± 0.07	93.73 ± 0.29	97.42 ± 0.04

**Table 4:** Hygroscopicity of DMY, CIP, and cocrystal under different humidity conditions.

Condition	Percentage of Weight Gain (%)		
	DMY	CIP	DMY-CIP
75% RH for 30d	1.10 ± 0.10	2.29 ± 0.15	1.39 ± 0.21
92.5% RH for 7d	0.76 ± 0.20	73.99 ± 4.74	5.73 ± 0.59

**Table 5:** Pharmacokinetics parameter after i.g in mice (x ± s, *n* = 6).

Parameter	Unit	Mixture	Cocrystal
AUC_(0-t)_	mg·L^−1^·h	0.871 ± 0.236	7.925 ± 1.295 ^#^
AUC_(0-∞)_	mg·L^−1^·h	1.016 ± 0.286	8.384 ± 1.2 ^#^
AUMC_(0-t)_	h·h·mg·L^−1^	0.491 ± 0.378	7.224 ± 2.287 ^#^
AUMC_(0-∞)_	h·h·mg·L^−1^	0.958 ± 0.895	9.026 ± 2.158 ^#^
MRT_(0-t)_	h	0.534 ± 0.316	0.892 ± 0.148 ^#^
MRT_(0-∞)_	h	0.847 ± 0.614	1.064 ± 0.131 ^*^
t_1/2z_	h	0.568 ± 0.372	0.738 ± 0.375 ^*^
T_max_	h	0.111 ± 0.068	0.278 ± 0.245 ^*^
CLz/F	L·h^−1^·kg^−1^	160.999 ± 59.206	18.229 ± 2.839 ^#^
Vz/F	L·kg^−1^	115.876 ± 53.684	19.796 ± 11.592 ^#^
C_max_	mg·L^−1^	2.017 ± 0.65	9.71 ± 3.015 ^#^

^#^ *p* < 0.05, ^*^ *p* > 0.05 compared with mixture group.

**Table 6:** Inhibition zone diameters of DMY, CIP, mixture, and cocrystal.

Bacterial Strains	Diameter of Inhibition Zone (cm)				
	Blank	DMY	CIP	Mixture	Cocrystal
*S. aureus*	0	0	1.50 ± 0.00	1.43 ± 0.06	1.47 ± 0.06 ^*^
*E. coli*	0	0	2.57 ± 0.06	2.53 ± 0.06	2.53 ± 0.06 ^*^

^*^ *p* > 0.05 compare with mixture and CIP group.

**Table 7:** MIC of DMY, CIP, mixture, and cocrystal.

Bacterial Strains	MIC (μg/mL)			
	DMY	CIP	Mixture	Cocrystal
*S. aureus*	312.5	1.0 ± 0.00	1.0 ± 0.00	0.83 ± 0.29 ^*^
*E. coli*	625	0.125 ± 0.11	0.083 ± 0.04	0.125 ± 0.11 ^*^

^*^ *p* > 0.05 compare with mixture and CIP group.

## Data Availability

The crystal structure has been included in Cambridge Crystallographic Data Centre (CCDC) with the number 2476323.

## Supplementary Materials

The following supporting information can be downloaded at: https://www.mdpi.com/article/10.3390/pharmaceutics17091209/s1, Figure S1. (a) b axis direction stacking projection of DMY-CIP cocrystal, (b) c axis direction stacking projection of DMY-CIP cocrystal; Figure S2. PXRD patterns of the remaining powder after solubility and dissolution experiments; Figure S3. Antibacterial effect of drugs on *Staphylococcus aureus* (From the bottom left clockwise were blank, dihydromyricetin, ciprofloxacin, mixed, cocrystal paper); Figure S4. Antibacterial effect of drugs on *Escherichia coli* (from the lower right clockwise were blank, dihydromyricetin, ciprofloxacin, mixed, cocrystal paper).

## Abbreviations

The following abbreviations are used in this manuscript:

API	Active Pharmaceutical Ingredients
BCS	Biopharmaceutics Classification System
