# Supplementary material for: Salt Cocrystallization—A Method to Improve Solubility and Bioavailability of Dihydromyricetin
Source: Pharmaceutics. 2025 Sep 17;17(9):1209. doi: 10.3390/pharmaceutics17091209 (PMC12473929; doi:10.3390/pharmaceutics17091209)
Supplement: Supplementary file 1 [file pharmaceutics-17-01209-s001.zip › pharmaceutics-3859672-supplementary.pdf]

Supplementary Materials for Salt Cocrystallization — a Method to Improve Solubility and Bioavailability of dihydromyricetin

Jingping Li, Xinke Chen, Yanan Liu and Caiwu Jiang *

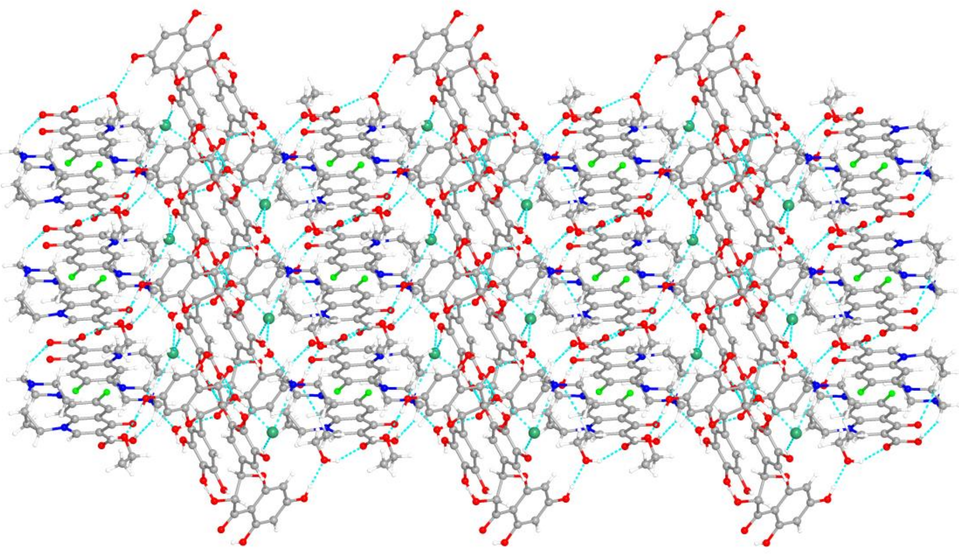

(a)

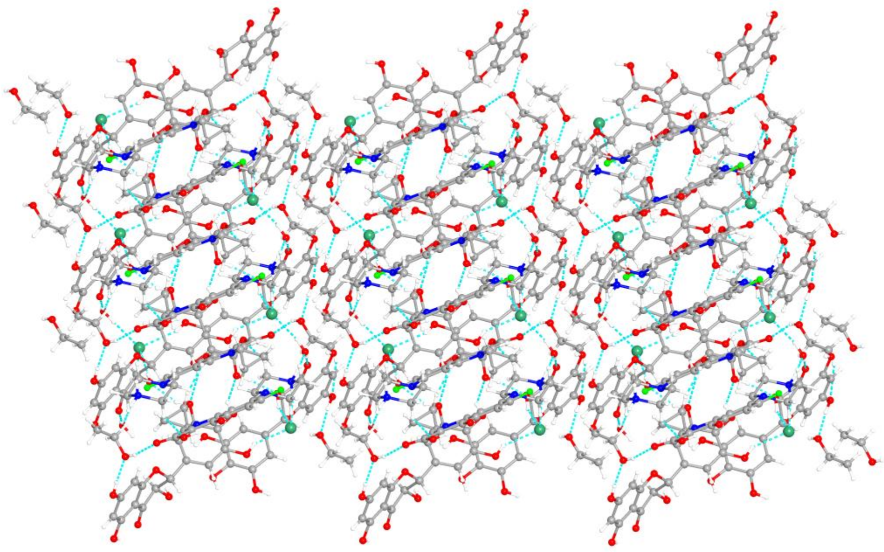

(b)

Figure S1. (**a**) b axis direction stacking projection of DMY-CIP cocrystal, (**b**) c axis direction stacking projection of DMY-CIP cocrystal.

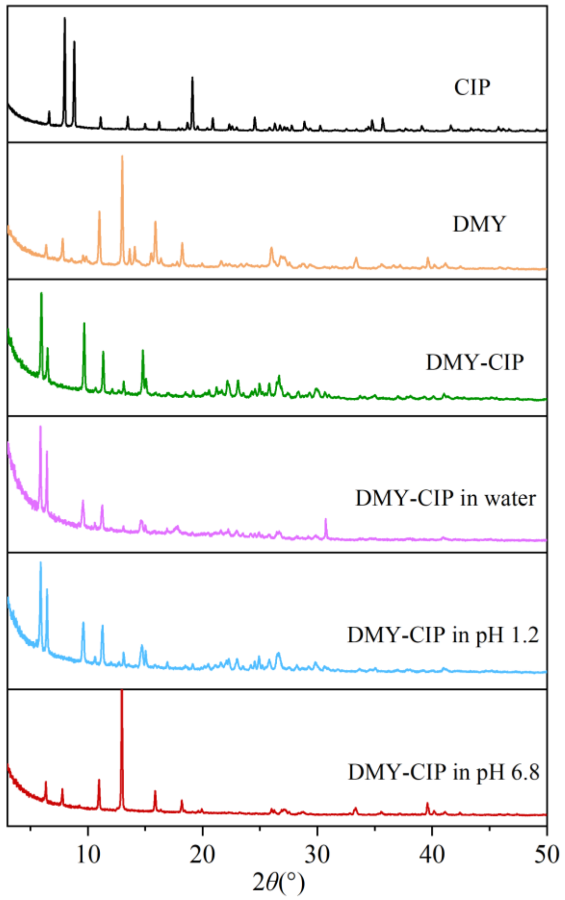

Figure S2. PXRD patterns of the remaining powder after solubility and dissolution experiments.

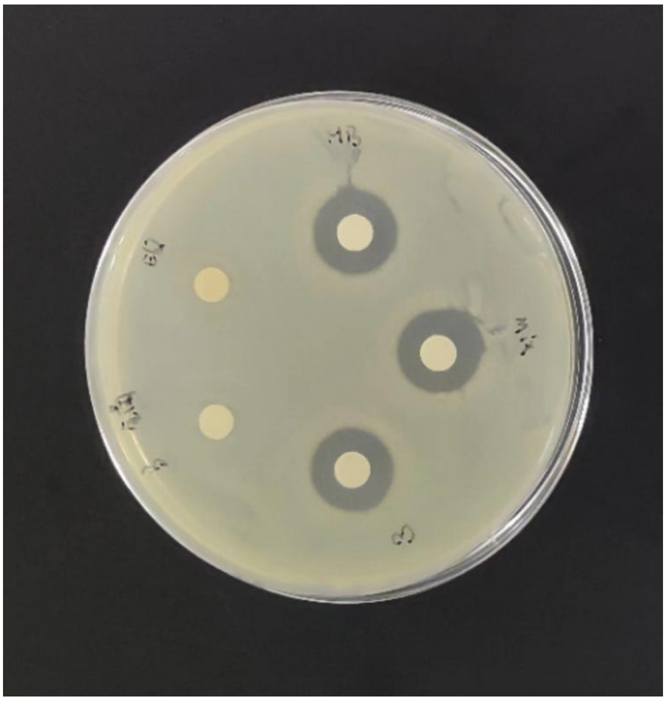

Figure S3. Antibacterial effect of drugs on *Staphylococcus aureus* (from the bottom left clockwise were blank, dihydromyricetin, ciprofloxacin, mixed, cocrystal paper).

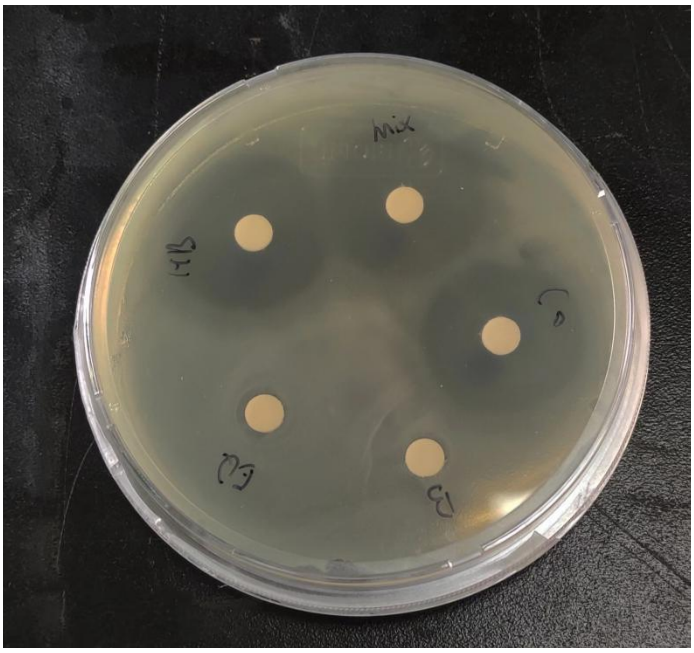

Figure S4. Antibacterial effect of drugs on *Escherichia coli* (from the lower right clockwise were blank, dihydromyricetin, ciprofloxacin, mixed, cocrystal paper).
